# E-cigarette use among university students in Vietnam following the 2025 e-cigarette ban policy: A multicity cross-sectional survey

**DOI:** 10.18332/tid/231598

**Published:** 2026-09-23

**Authors:** Thi Thanh Huong Le, Bich Thao Nguyen Thi, Xuan Quy Luu, Anh Quynh Nguyen, Thi Thu Trang Phan, Thi Hoa Mai Tran, Hoang Minh Dao Le, Thi Thu Huyen Doan, Minh Tan Luong

**Affiliations:** 1 Faculty of Environmental and Occupational Health, Hanoi University of Public Health, Hanoi, Vietnam; 2 Faculty of Fundamental Sciences, Hanoi University of Public Health, Hanoi, Vietnam; 3 National Cancer Center Graduate School of Cancer Science and Policy, Goyang, Korea; 4 Campaign for Tobacco Free Kids, Vietnam Office Hanoi, Vietnam

**Keywords:** e-cigarettes, university students, associated factors, multicity survey, Vietnam

## Abstract

**INTRODUCTION:**

Electronic cigarette (e-cigarette, EC) use is a growing public health concern among young people worldwide. In Vietnam, Resolution No. 173/2024/QH15 prohibited e-cigarettes and heated tobacco products from January 2025. However, evidence on EC use among university students after implementation of this policy remains limited. This study assessed the prevalence of EC use and associated factors among Vietnamese university students in the post-ban period.

**METHODS:**

A cross-sectional study was conducted among 2462 students recruited using a multi-stage cluster sampling approach from universities in Hanoi, Hue, Da Nang, and Ho Chi Minh City representing major urban regions, between October and December 2025. Data were collected using a structured self-administered questionnaire. Multivariable logistic regression, adjusted for sociodemographic and social/behavioral covariates, was performed to identify factors associated with e-cigarette use.

**RESULTS:**

The mean age of the study participants was 19.5 years (SD=1.3). The prevalence of ever use (defined as former and current use) and current e-cigarette use was 17.7% and 3.3%, respectively. Female students were less likely than males to report ever use (adjusted odds ratio, AOR=0.70; 95% CI: 0.51–0.96) and current use (AOR=0.30; 95% CI: 0.13–0.72). Ever e-cigarette use was associated with peer use of e-cigarettes or heated tobacco products (AOR=3.17; 95% CI: 2.40–4.19), conventional cigarette smoking (AOR=21.00; 95% CI: 15.74–28.04), and exposure to e-cigarette advertising (AOR=1.36; 95% CI: 1.03–1.79). Current use was associated with family member use (AOR=2.62; 95% CI: 1.17–5.87), peer use (AOR=3.35; 95% CI: 1.92–5.87), conventional cigarette smoking (AOR=14.83; 95% CI: 7.91–27.83), and exposure to e-cigarette advertising (AOR=2.49; 95% CI: 1.47–4.21).

**CONCLUSIONS:**

Although current e-cigarette use was lower than levels reported in several pre-ban university studies, use remains. Male students, conventional cigarette smokers, students with peers or family members using ECs or heated tobacco products, and those exposed to advertising were more likely to report use. Continued surveillance and further longitudinal studies are needed to monitor EC use trends and to evaluate the long-term impact of the ban among Vietnamese university students.

## INTRODUCTION

Electronic cigarettes (e-cigarettes or ECs) are battery-operated devices that heat a liquid solution, typically containing nicotine, flavorings, and other chemicals, to generate an aerosol inhaled by users^1^. Although ECs do not involve tobacco combustion, they are not harmless. According to the World Health Organization (WHO), EC aerosols contain nicotine, ultrafine particles, heavy metals, volatile organic compounds, and carcinogenic compounds that may adversely affect human health^1,2^. Evidence has linked EC use with respiratory inflammation, impaired immune responses, cardiovascular dysfunction, and nicotine dependence, particularly among adolescents and young adults whose brains are still developing^2-4^. Consequently, ECs should not be regarded as harmless alternatives to combustible tobacco products, especially among youth populations^5^. Heated tobacco products (HTPs), which heat processed tobacco rather than a liquid solution are another emerging nicotine product with similar patterns of use and health concerns among young people^6^, and were included alongside ECs under Vietnam’s 2025 prohibition policy^7^.

Despite increasing evidence regarding health risks, misconceptions about ECs remain common among adolescents and young adults. Many perceive ECs as less harmful alternatives to conventional cigarettes (CC) or as effective smoking cessation aids. Such perceptions are associated with a greater likelihood of experimentation and continued use. International studies have reported increasing EC use among young people, raising concerns regarding nicotine dependence, dual use, and subsequent transitions to combustible tobacco smoking^5^.

Prior to the implementation of the nationwide ban on ECs and HTPs in 2025, EC use had become an emerging public health concern in Vietnam. A recent study reported that 7.8% of Vietnamese adolescents had ever used EC and 3.5% were current users, compared with 1.5% and 0.6%, respectively, for heated tobacco products (HTPs)^8^. Among university students, current EC use reached 13.2% in Hanoi^9^, while another study of Vietnamese young adults reported an overall prevalence of 8.35% and found that perceiving ECs as less harmful was associated with a greater likelihood of use^10^. More recently, a multi-university survey conducted in Vietnam found that 9.0% of students were current EC users^11^. National data also indicated a prevalence of 2.6% among in-school adolescents in 2019^12^. Previous research further identified peer and parental smoking and exposure to EC/HTP advertising as factors associated with EC use among Vietnamese adolescents and young people^13,14^. Collectively, these findings suggest that ECs had become increasingly common among Vietnamese youth before the nationwide prohibition policy. Internationally, regulatory approaches to ECs vary, ranging from restrictions on their sale, advertising, and use to complete sale bans. According to the WHO Report on the Global Tobacco Epidemics 2025, 42 countries had banned the sale of electronic nicotine delivery systems (ENDS), while many others had adopted regulatory measures to control these products^15^. Vietnam’s nationwide prohibition therefore forms part of a broader global regulatory response to the public health concerns associated with ECs.

In response to the growing public health concern surrounding ECs and other novel tobacco products, the Vietnamese National Assembly adopted Resolution No. 173/2024/QH15 on 30 November 2024, which came into effect on 1 January 2025. The resolution prohibits the production, importation, trading, transportation, storage, and commercial distribution of e-cigarettes and heated tobacco products nationwide^7^. While this policy represents a major milestone in tobacco control, evidence regarding EC use after its implementation remains limited.

To date, most studies on EC use in Vietnam have been conducted in the pre-ban period. Evidence on the prevalence of EC use among university students following the implementation of the national prohibition policy is still lacking. To address this gap, this cross-sectional study aimed to determine the prevalence of ever and current EC use among university students, and to identify factors associated with EC use in the post-ban context.

## METHODS

### Study design and setting

Data used in this study were extracted from a cross-sectional multicity survey conducted between October and December 2025 among university students. The study was implemented in three major regions: Hanoi, representing northern Vietnam; Hue and Da Nang, representing central Vietnam; and Ho Chi Minh City, representing southern Vietnam.

### Study population and sampling

A multi-stage clustered sampling approach was used. In the first stage, the three study regions described above (Hanoi, Hue and Da Nang, and Ho Chi Minh City) were selected as the primary sampling units. In each study area, four universities or colleges were purposively selected for survey implementation based on institutional willingness to participate and diversity of academic disciplines offered across technical, social, and health sciences. Within each selected university or college, classes were randomly selected by academic year. For each year of study, two classes were randomly selected from first-year, second-year, third-year, and fourth-year classes. Within each selected class, approximately 25 eligible students were randomly selected and invited to participate in the survey. This procedure was used to obtain representation across institutions and academic years within each study area. Students were eligible to participate if they were Vietnamese nationals officially enrolled at the selected university or college, present in the randomly selected class on the day of data collection, and willing to provide informed consent. Data for the present study were drawn from a broader cross-sectional survey conducted in 2025 to assess compliance with regulations on e-cigarettes and heated tobacco products and exposure to advertising and promotion of these products. The minimum required sample size for the original survey was calculated based on an expected prevalence of 73.3% for exposure to e-cigarette advertising^14^, with a 95% confidence level and a precision of 5%, resulting in a minimum sample size of 2190 students (730 students per region). During the data collection, 2485 students participated in the study. Of these, 23 students did not complete the questionnaire and were excluded from the analysis. The final analytic sample included 2462 students aged 18–24 years, who were enrolled at universities or colleges in the selected study areas.

### Measurements

Data were collected using an anonymous, self-administered questionnaire in Vietnamese. The questionnaire was developed with reference to items from the 2023 Global Youth Tobacco Survey^16^ and the 2025 National Youth Tobacco Survey^17^, with adaptation for the Vietnamese university student context and for questions related to new tobacco products. It was piloted with 30 students at universities in Hanoi that were not included in the main study. Based on the pilot results, item wording was clarified, and response options for product-use items were refined before the data collection. The questionnaire was translated into Vietnamese and back-translated into English by two investigators in the research team to ensure conceptual and semantic equivalence with the source instruments. The questionnaire items used to derive the variables included in the present analysis are provided in the Supplementary file.

### Outcome variables

The main outcome was self-reported EC use status. Participants were classified as never users, former users, or current users of ECs. Ever EC use was defined as reporting former or current EC use, compared with never use. Current EC use was defined as reporting current EC use, compared with non-current use, including never and former use.

### Independent variables

Exposure to EC and HTP advertisements during the past 30 days was self-reported. Advertising exposure was defined as contact with any message, activity, or promotional content related to these products within the 30 days before the survey^18^.

Participants were asked where they had seen advertisements for e-cigarettes or heated tobacco products in the past 30 days. Advertising channels included social media, internet or websites, posters or displays at retail stores, and events. For the main analysis, advertising exposure was coded as a binary variable: any exposure to EC or HTP advertising in the past 30 days versus no exposure.

### Potential covariates

Other variables included sociodemographic and academic characteristics as well as social and behavioral variables. Sociodemographic and academic variables included age, sex, university year, field of study, study region, and living arrangement. Age was not entered as a separate covariate because of its high collinearity with university year, and the sample age range (18–24 years) is reported under Study population and sampling. Sex was categorized as male or female. Student university year was categorized as first and second year versus third year and higher. Field of study was grouped as Economics/Communication/Marketing, Engineering/Language/Education, and Health Sciences; this three-category classification, rather than a binary health/non-health grouping, was used to capture potential heterogeneity in health literacy and risk perception across broad academic disciplines beyond the health field alone. Study region was categorized as Hanoi, Hue and Da Nang, and Ho Chi Minh City. Living arrangement was categorized as living with parents or relatives versus living alone or with a friend in a dormitory, rented housing, or other accommodation. These sociodemographic, academic, social, and behavioral covariates were selected based on their established associations with EC use in previous studies among Vietnamese and international youth populations^13,14,19-21^.

Social and behavioral covariates included having a family member who used ECs or HTPs, having friends who used ECs or HTPs, and CC smoking status. Family and friend use of ECs or HTPs were coded as a binary variable (yes, no). The two products were combined within these measures because young people may not consistently distinguish between ECs and HTPs^22-24^, and both products are subject to the same regulatory framework in Vietnam^7^.

### Statistical analysis

Descriptive statistics were used to summarize participant characteristics. Categorical variables are presented as frequencies and percentages. Missing data on covariates were handled using an available case approach and excluded from the relevant analyses on a variable-by-variable basis; the number of missing observations for each variable is reflected in the total reported in Table 1. Differences in participant characteristics by e-cigarette use status were assessed using chi-squared tests.

**Table 1:** Demographic characteristics and EC use status among the student participants at Hanoi, Hue, Da Nang, and Ho Chi Minh cities, October–December 2025 (N=2462)

*Characteristics*	*Total* *n (%)*	*Male* *n (%)*	*Female* *n (%)*	*p*
**Total**	2462 (100)	1352 (54.9)	1110 (45.1)	
**Age** (years), mean (SD)	19.5 (1.3)	19.5 (1.4)	19.4 (1.2)	0.062
**University year**				0.47
First and second	1456 (59.2)	790 (58.6)	666 (60.0)	
Third year and higher	1003 (40.8)	559 (41.4)	444 (40.0)	
**Field of study**				**<0.001**
Economics/Communication/Marketing	844 (34.3)	332 (24.6)	512 (46.1)	
Engineering/Language/Education	1156 (47.0)	807 (59.7)	349 (31.4)	
Health Sciences	462 (18.8)	213 (15.8)	249 (22.4)	
**Study region**				**0.003**
Hanoi	853 (34.6)	467 (34.5)	386 (34.8)	
Hue and Da Nang	810 (32.9)	480 (35.5)	330 (29.7)	
Ho Chi Minh City	799 (32.5)	405 (30.0)	394 (35.5)	
**Living arrangement**				0.50
Living with parents/relatives	1192 (48.4)	663 (49.0)	529 (47.7)	
Living alone or with friends/colleagues	1270 (51.6)	689 (51.0)	581 (52.3)	
**Family member using ECs/HTPs**				0.69
No	2290 (93.0)	1255 (92.8)	1035 (93.2)	
Yes	172 (7.0)	97 (7.2)	75 (6.8)	
**Friends using ECs/HTPs**				**<0.001**
No	1662 (67.5)	840 (62.1)	822 (74.1)	
Yes	800 (32.5)	512 (37.9)	288 (25.9)	
**Exposure to EC/HTP advertising**				0.36
No	1523 (61.9)	825 (61.1)	698 (62.9)	
Yes	938 (38.1)	526 (38.9)	412 (37.1)	

P-values estimated using chi-squared tests. Totals may vary because of missing data. Gender was not tested against itself. HTPs: heated tobacco products. EC: e-cigarette.

Logistic regression models were used to assess factors associated with EC use status. Separate models were fitted for two outcomes: ever EC use and current EC use. Crude odds ratios (ORs) and adjusted odds ratios (AORs) with 95% confidence intervals (CIs) are reported. Multivariable models were adjusted for sociodemographic characteristics and social and behavioral covariates, wherein variables that were statistically significant in the univariable model were included in the multivariable model in addition to the demographic variables. All analyses were conducted using Stata version 18.0. A p<0.05 was considered statistically significant.

### Ethical considerations

The study protocol was reviewed and approved by the Ethics Committee of the Hanoi University of Public Health under Decision number 288/2025/YTCC-HD3 dated 6 June 2025. Before the survey, students received an information sheet and provided informed consent. Students who did not wish to participate were free to decline, and non-participation did not affect their academic rights or benefits. The questionnaire did not collect names or directly identify personal information. All data were used only for research purposes and were stored securely to protect participant confidentiality.

## RESULTS

### Demographic characteristics and EC use status among the student participants

The demographic characteristics and the use of ECs among university students involved in the study are presented in Table 1. A total of 2462 university students participated in the study, of whom 54.9% were male and 45.1% were female. Nearly three-fifths (59.2%) were first- or second-year students, with a mean age of 19.5 years (SD=1.3), and 47.0% were studying engineering, languages, or education. Participants were relatively evenly distributed across Hanoi (34.6%), Hue and Da Nang (32.9%), and Ho Chi Minh City (32.5%). Approximately half of the study participants lived with parents or relatives (48.4%), while 51.6% lived in dormitories, rented accommodation, or other settings. Overall, 7.0% reported having a family member and 32.5% reported having friends who used ECs or HTPs. Around 38.1% of students were exposed to EC/HTP advertising and promotion. Significant differences by sex were observed in field of study, study region, and friends using ECs or HTPs (all p<0.05); male students were more likely to study engineering/language/education and to report friends who use ECs or HTPs; while the regional distribution also differed significantly by sex (Table 1).

### Prevalence of EC use among the student participants

Table 2 presents the prevalence of CC, EC, and HTP use among university students. Among the three product categories examined, CCs were the most used, with 18.6% of students reporting ever use. ECs were the second most commonly used product, with an ever use prevalence of 17.7% and a current use prevalence of 3.3%. HTP use was less common, with 7.0% reporting ever use. Male students consistently reported higher prevalence than females across all product types (all p<0.001) (Table 2). The sex gap was particularly pronounced for EC use, where current use among males (5.5%) was approximately nine times higher than among female students (0.6%).

**Table 2:** Tobacco products and EC use status among student participants, by sex (N=2462)

*Characteristics*	*Total* *n (%)*	*Male* *n (%)*	*Female* *n (%)*	*p*
**EC use status**				**<0.001**
Never	2027 (82.3)	1008 (74.6)	1019 (91.8)	
Former	353 (14.3)	269 (19.9)	84 (7.6)	
Current	82 (3.3)	75 (5.5)	7 (0.6)	
**CC smoking**				**<0.001**
Never	2004 (81.4)	950 (70.3)	1054 (95.0)	
Ever	458 (18.6)	402 (29.7)	56 (5.0)	
**HTP use**				**<0.001**
Never	2289 (93.0)	1204 (89.1)	1085 (97.7)	
Ever	173 (7.0)	148 (10.9)	25 (2.3)	

Percentages were calculated within each sex group. Ever EC use was defined as reporting former or current EC use, compared with never use. Current EC use was defined as reporting current EC use, compared with non-current use, including never and former use. Ever EC use (former and current use combined) was reported by 17.7% (435/2462) of participants. EC: e-cigarette. CC: conventional cigarette. HTP: heated tobacco product. P-values calculated using chi-squared tests.

### Factors associated with ever EC use among the student participants

The multivariable logistic regression analysis of 2462 university students identified several significant factors for having ever used ECs (Table 3). Sex remained a significant factor, with female students showing 30.0% lower odds of ever use compared to males (adjusted odds ratio, AOR=0.70; 95% CI: 0.51–0.96, p<0.05). A history of CC smoking was the factor most strongly associated with ever EC use, increasing the odds of ever using ECs by 21 times (AOR=21.00; 95% CI: 15.74–28.04, p<0.01).

**Table 3:** Univariable and multivariable analyses of factors associated with ever EC use among the student participants (N=2462)

*Variables*	*Never use* *n (%)*	*Ever use* *n (%)*	*OR (95% CI), p*	*AOR (95% CI), p*
**Sex**				
Male (ref.)	1008 (49.7)	344 (79.1)	1.00	1.00
Female	1019 (50.3)	91 (20.9)	0.26 (0.20–0.33), <0.01**	0.70 (0.51–0.96), <0.05*
**University year**				
First and second (ref.)	1238 (61.1)	218 (50.3)	1.00	1.00
Third year and higher	788 (38.9)	215 (49.7)	1.55 (1.26–1.91), <0.01**	1.26 (0.95–1.68)
**Field of study**				
Economics/Communication/Marketing (ref.)	692 (34.1)	152 (34.9)	1.00	1.00
Engineering/Language/Education	926 (45.7)	230 (52.9)	1.13 (0.90–1.42)	0.88 (0.64–1.20)
Health Sciences	409 (20.2)	53 (12.2)	0.59 (0.42–0.83), <0.01**	0.72 (0.46–1.12)
**Study region**				
Hanoi (ref.)	673 (33.2)	180 (41.4)	1.00	1.00
Hue and Da Nang	668 (33.0)	142 (32.6)	0.79 (0.62–1.02)	1.10 (0.78–1.56)
Ho Chi Minh City	686 (33.8)	113 (26.0)	0.62 (0.48–0.80), <0.01**	1.29 (0.89–1.86)
**Living arrangement**				
Living with parents/relatives (ref.)	989 (48.8)	203 (46.7)	1.00	
Living alone or with friends/colleagues	1038 (51.2)	232 (53.3)	1.09 (0.88–1.34)	
**Family member using EC/HTPs**				
No (ref.)	1882 (92.8)	408 (93.8)	1.00	
Yes	145 (7.2)	27 (6.2)	0.86 (0.56–1.31)	
**Friends using EC/HTPs**				
No (ref.)	1477 (72.9)	185 (42.5)	1.00	1.00
Yes	550 (27.1)	250 (57.5)	3.63 (2.93–4.49), <0.01**	3.17 (2.40–4.19), <0.01**
**CC smoking**				
Never (ref.)	1865 (92.0)	139 (32.0)	1.00	1.00
Ever	162 (8.0)	296 (68.0)	24.52 (18.95–31.72), <0.01**	21.00 (15.74–28.04), <0.01**
**Exposure to EC/HTP advertising**				
No (ref.)	1290 (63.7)	233 (53.6)	1.00	1.00
Yes	736 (36.3)	202 (46.4)	1.52 (1.23–1.87), <0.01**	1.36 (1.03–1.79), <0.01**

AOR: adjusted odds ratio. Adjusted models included sociodemographic characteristics and social, behavioral and marketing covariates. Ever EC use was defined as reporting former or current use, compared with never use. Current EC use was defined as reporting current EC use, compared with non-current use, including never and former use. CI: confidence interval. EC: e-cigarette. CC: conventional cigarette. HTP: heated tobacco product. P-values calculated using chi-squared tests.

** p<0.01,

* p<0.05.

Hosmer-Lemeshow goodness-of-fit test p=0.7284; AUC=0.8644.

Social and environmental factors were also associated with ever EC use among the study participants. Students with friends who use EC or HTPs were over three times more likely to have ever experimented with EC use (AOR=3.17; 95% CI: 2.40–4.19, p<0.01). Additionally, exposure to EC/HTP advertisements was associated with a 36.0% increase in the likelihood of ever using EC (AOR=1.36; 95% CI: 1.03–1.79, p<0.01). Interestingly, while third-year students and those in health sciences showed significant associations in the univariable analysis, these factors were no longer significant after adjusting for other variables in the multivariable model (Table 3).

### Factors associated with current EC use among the student participants

Table 4 represents the associations with current use of ECs among the student participants. Female students were significantly less likely than male students to be current EC users (AOR=0.30; 95% CI: 0.13–0.72, p<0.01). Regional differences emerged as an associated factor for EC use, with students in Hue and Da Nang (AOR=0.46; 95% CI: 0.24–0.90) and Ho Chi Minh City (AOR=0.45; 95% CI: 0.21–0.95) being significantly less likely to be current EC users compared to those in Hanoi city.

**Table 4:** Univariable and multivariable analyses of factors associated with current EC use among the student participants (N=2462)

*Variables*	*Non-current* *use* *n (%)*	*Current use* *n (%)*	*OR (95% CI), p*	*AOR (95% CI), p*
**Sex**				
Male (ref.)	1277 (53.7)	75 (91.5)	1.00	1.00
Female	1103 (46.3)	7 (8.5)	0.11 (0.05–0.24), <0.01**	0.30 (0.13–0.72), <0.01**
**University year**				
First and second (ref.)	1415 (59.5)	41 (50.0)	1.00	1.00
Third year and higher	962 (40.5)	41 (50.0)	1.47 (0.95–2.29)	0.76 (0.43–1.35)
**Field of study**				
Economics/Communication/Marketing (ref.)	809 (34.0)	35 (42.7)	1.00	1.00
Engineering/Language/Education	1112 (46.7)	44 (53.7)	0.91 (0.58–1.44)	0.75 (0.44–1.30)
Health Sciences	459 (19.3)	3 (3.7)	0.15 (0.05–0.49), <0.01**	0.38 (0.11–1.38)
**Study region**				
Hanoi (ref.)	801 (33.7)	52 (63.4)	1.00	1.00
Hue and Da Nang	793 (33.3)	17 (20.7)	0.33 (0.19–0.58)	0.46 (0.24–0.90), <0.01**
Ho Chi Minh City	786 (33.0)	13 (15.9)	0.25 (0.14–0.47)	0.45 (0.21–0.95), <0.01**
**Living arrangement**				
Living with parents/relatives (ref.)	1150 (48.3)	42 (51.2)	1.00	
Living alone or with friends/colleagues	1230 (51.7)	40 (48.8)	0.89 (0.57–1.38)	
**Family member using ECs/HTPs**				
No (ref.)	2220 (93.3)	70 (85.4)	1.00	1.00
Yes	160 (6.7)	12 (14.6)	2.38 (1.26–4.48), <0.01**	2.62 (1.17–5.87), <0.05*
**Friends using ECs/HTPs**				
No (ref.)	1642 (69.0)	20 (24.4)	1.00	1.00
Yes	738 (31.0)	62 (75.6)	6.90 (4.14–11.50), <0.01**	3.35 (1.92–5.87), <0.01**
**CC smoking**				
Never (ref.)	1990 (83.6)	14 (17.1)	1.00	1.00
Ever	390 (16.4)	68 (82.9)	24.78 (13.80–44.50), <0.01**	14.83 (7.91–27.83), <0.01**
**Exposure to EC/HTP advertising**				
No (ref.)	1496 (62.9)	27 (32.9)	1.00	1.00
Yes	883 (37.1)	55 (67.1)	3.45 (2.16–5.51), <0.01**	2.49 (1.47–4.21), <0.01**

AOR: adjusted odds ratio. Adjusted models included sociodemographic characteristics and social, behavioral and marketing covariates. Ever EC use was defined as reporting former or current use, compared with never use. Current EC use was defined as reporting current EC use, compared with non-current use, including never and former use. CI: confidence interval. EC: e-cigarette. CC: conventional cigarette. HTP: heated tobacco product. P-values were calculated using chi-squared tests.

** p<0.01,

* p<0.05.

Hosmer-Lemeshow goodness-of-fit test p=0.7144; AUC=0.9195.

The social environment was also associated with current EC use. Unlike ever use, living with a family member who uses ECs/HTPs was significantly associated with current EC use, increasing the odds by 2.6 times (AOR=2.62; 95% CI: 1.17–5.87, p<0.05). Peer influence remained significantly associated with current EC use, with those having friends who use ECs or HTPs being over three times more likely to be current EC users (AOR=3.35; 95% CI: 1.92–5.87, p<0.01) compared with those without friends who used ECs or HTPs.

Finally, behavioral and marketing influences were significant with EC use among the student participants. A history of CC smoking increased the odds of current EC use by nearly 15 times (AOR=14.83; 95% CI: 7.91–27.83, p<0.01). Furthermore, students exposed to EC/HTP advertisement for these products had approximately 2.5 times higher odds of being current EC users (AOR=2.49; 95% CI: 1.47–4.21, p<0.01), compared with those not exposed to advertising (Table 4).

## DISCUSSION

### The prevalence of ever use and current use of ECs among the student participants

This study found that 17.7% of university students had ever used ECs, while 3.3% were current users. The prevalence of ever use was higher than that reported in a previous study conducted in Hanoi and Ho Chi Minh City (7.4%)^14^ but lower than the 20.4% observed among engineering students in Hanoi city^9^. Variations in study populations may partly explain these differences. The former study included both adolescents and young adults aged 15–24 years, whereas the present study was limited to university students. Meanwhile, the latter study focused exclusively on engineering students, who may have different risk profiles and patterns of EC use compared with students from a broader range of academic disciplines.

In contrast, the prevalence of current EC use observed in the present study was substantially lower than that reported in studies conducted before the implementation of the national ban (from 1 January 2025). A survey of university students conducted in 2023 reported a current EC use prevalence of 9.0%^11^, while a previous study in Hanoi city found that 13.2% of students were current EC users^9^. Notably, the prevalence observed in the present study (3.3%) was also similar to that reported among Vietnamese adolescents in the 2022 Global Youth Tobacco Survey (GYTS) (3.5%)^8^, despite the present student participants being older than those observed in the GYTS study. This may explain the higher prevalence of EC use among young adults than adolescents. The markedly lower prevalence observed in the present study may also reflect reduced product availability, accessibility, and social acceptability following the nationwide prohibition of ECs and HTPs under Resolution No. 173/2024/QH15, which came into effect in January 2025^7^. As data collection was conducted during the final quarter of 2025, this study’s findings provide some of the earliest empirical evidence on EC use among Vietnamese university students in the post-ban period.

Nevertheless, caution is warranted when interpreting these differences, as variations in study settings, sampling strategies, and participant characteristics may also contribute to the observed disparities in prevalence estimates. Further monitoring is needed to determine whether the reduction in current EC use can be sustained over time as well as to assess the long-term impact of the national ban on EC use behaviors among young adults in Vietnam, especially among university students.

### Factors associated with ever use and current use of ECs among the student participants

The univariable and multivariable logistic regression analyses identified several factors associated with both ever and current EC use among university students in Vietnam. Female students were significantly less likely than male students to report both ever use and current use of ECs. Similar sex disparities have been consistently reported among Vietnamese university students and in studies from other countries^9,11,19-21,25-27^. This pattern may reflect higher engagement in risk-taking and substance-related behaviors among males, as well as greater peer influences and more favorable social norms toward smoking and EC use^19-21^.

Family influences were also associated with EC use. Students with family members who used ECs or HTPs were more likely to report both ever and current EC use. Similar findings have been reported in Vietnam^9,13^ and internationally^27,28^. Family members may serve as important behavioral models, and young people are more likely to imitate tobacco- and nicotine-related behaviors observed within the household^28^. This finding suggests a potential role of the family environment in EC use among university students, although causal inference cannot be drawn from this cross-sectional design.

Peer influence emerged as a factor associated with EC use. Students who reported having friends who used ECs or HTPs were more likely to have ever used ECs and to be current EC users. This finding is consistent with previous evidence among Vietnamese adolescents^8^ and international studies^27-29^. Peer networks may facilitate EC use through social modeling, product accessibility, and the normalization of nicotine use within friendship groups^29^. Interestingly, this association was not observed in two earlier studies among Vietnamese university students^9,11^, suggesting that peer influence may have become increasingly important as EC use has become more common among young people in recent years^8,28^.

CC smoking was another factor associated with EC use among university students. Students who smoked CCs were significantly more likely to have ever used and currently use ECs than non-smokers. This finding is consistent with previous studies in Vietnam^8,9,13^ and internationally^21^. A recent global systematic review and meta-analysis reported that CC smokers were approximately five times more likely to use ECs than non-smokers^21^. This association may partly reflect shared misconceptions among smokers that ECs are a less harmful alternative to combustible cigarettes or an effective aid for smoking cessation, despite limited and inconsistent evidence supporting the latter claim^3,5^ as well as a general tendency for EC use to co-occur with other tobacco- and nicotine-related behaviors^10,11,21^.

Exposure to EC/HTP advertising through multiple channels, including retail outlets and online platforms, was also significantly associated with both ever and current EC use in this study. This finding is consistent with evidence from Vietnam^14,30^ and other countries^27,31,32^, suggesting that exposure to EC/HTP advertising may increase product awareness, reduce perceived harms, and enhance the social acceptability of EC use among young people. Notably, the persistence of this association in the post-ban period may indicate continued exposure to EC/HTP advertising and promotional content through online and informal channels. Although Resolution No. 173/2024/QH15 also restricts the advertising and marketing of ECs and HTPs, its enforcement may not fully extend to informal promotion through social media influencers or peer-to-peer sharing, which are increasingly common marketing channels among young people^33^. The persistence of advertising exposure in the post-ban period may also reflect continued availability of these products through illicit or online markets operating outside formal regulatory oversight, which requires further investigation and stronger cross-sectional enforcement.

Regional differences were also observed, with students in Hanoi reporting a higher prevalence of current EC use than those in Hue and Da Nang, and Ho Chi Minh City. Several contextual factors may plausibly contribute to this variation. Previous research in Hanoi and Ho Chi Minh City has documented substantial exposure of young people to EC advertising through multiple channels, particularly social media, and demonstrated an association between advertising exposure and EC use; and exposure to EC/HTP advertising varies between major urban centers in Vietnam^14^. Differences across cities in product availability, informal distribution networks, marketing exposure, and local implementation or enforcement of the national ban could therefore potentially influence access to and use of ECs^34,35^. Social and behavioral environments may also differ across university settings and cities^9,11,36^. However, these contextual factors were not directly measured in the present study, and previous Vietnamese studies have not provided sufficient evidence to determine why EC use would be higher in Hanoi than in the other study locations. The observed regional difference should therefore be considered an important finding requiring further investigation.

The strong association between CC smoking and EC use indicates substantial clustering of CC and EC use within this population. Despite the large effect estimate, its confidence interval (CI) was relatively precise, likely because CC smoking was sufficiently prevalent in the study population to provide an adequate number of observations for estimation. In contrast, the wider CI observed for family member EC/HTP use in relation to current EC use (AOR=2.62; 95% CI: 1.17–5.87) likely reflects the relatively small number of students reporting both the exposure and outcome. Only 7.0% of the participants reported having a family member who used ECs or HTPs, while current EC use itself was uncommon (3.0%). Consequently, fewer observations contributed information to this association, resulting in lower statistical precision and a wider CI. Although the association remained statistically significant, its magnitude should therefore be interpreted with appropriate caution.

### Strengths and limitations

This study is among the first multicity surveys to assess EC use among Vietnamese university students following implementation of the national ban on ECs and HTPs. The large sample size and inclusion of universities from northern, central, and southern Vietnam provided timely evidence on EC use in the post-ban context. In addition, the study examined a broad range of demographic, social, behavioral, and marketing factors associated with EC use, generating evidence that may inform future surveillance, prevention, and tobacco control policies in Vietnam.

However, several limitations should be considered when interpreting the findings of this study. First, data were based on self-reported information and may therefore be subject to recall or social desirability bias. Because the survey was conducted after the implementation of the nationwide ban on ECs and HTPs, some respondents may have been reluctant to disclose use of these prohibited products because of social desirability bias or concern about potential legal or academic consequences, which may have led to underreporting of true product use. Second, although the study included students from multiple universities across four major cities, the findings may not be fully generalizable to all university students in Vietnam. Third, as a cross-sectional design, this study cannot establish temporality between exposures and EC use, and reverse causation cannot be excluded; selection bias may also have occurred, as participation depended on students’ presence in class on the day of data collection and their willingness to complete the questionnaire, which may differ systematically between EC users and non-users. Fourth, although the questionnaire was adapted from WHO-based instruments using forward-backward translation and pretested for clarity and cultural appropriateness among 30 students outside the main study sample, formal psychometric validation (e.g. internal consistency or construct validity assessment) of the Vietnamese version was not conducted. Further validation should therefore be considered in future application of this instrument. Fifth, multicollinearity assessment, such as variance inflation factors, was not performed. Although covariates were selected *a priori* based on conceptual relevance and evidence from previous studies, some residual collinearity cannot be excluded. Finally, other potentially relevant factors, such as nicotine dependence, mental health conditions, detailed patterns of social media engagement, and exposure to illicit online sales channels, were not assessed and may have contributed to residual confounding. Future longitudinal studies are needed to better understand the evolving patterns of EC use and to evaluate the long-term impact of Vietnam’s nationwide ban on emerging tobacco products among young adults.

## CONCLUSIONS

EC use remained notable among Vietnamese university students in 2025 despite the nationwide ban on ECs and HTPs. Male sex, CC smoking, peer and family use of ECs or HTPs, and exposure to EC/HTP advertising were independently associated with both ever and current EC use. These findings suggest that social influences and continued exposure to promotional content remain potential drivers of EC use among university students in Vietnam. Ongoing surveillance and longitudinal research are needed to monitor trends in EC use and to evaluate the long-term impact of the national ban policy. Strengthened monitoring and control of EC/HTP advertising, marketing and sales, particularly through online channels, alongside university-based health education, may further reduce EC use among university students.

## Supplementary Material



## Data Availability

The data supporting this research are available from the authors on reasonable request.
