# Supplementary material for: E-cigarette use among university students in Vietnam following the 2025 e-cigarette ban policy: A multicity cross-sectional survey
Source: Tob Induc Dis. 2026 Sep 23;24:10.18332/tid/231598. doi: 10.18332/tid/231598 (PMC13602399; doi:10.18332/tid/231598)
Supplement: Supplementary file 1 [file TID-24-176-s1.pdf]

Tobacco Induced Diseases

Supplementary file

© 2026 Le T.T.H. et al.

DOI:
10.18332/tid/231598

The content has been provided by the author(s) and has not been reviewed, verified, or endorsed by European Publishing. It may not have undergone peer review. The views, opinions, and recommendations expressed are solely those of the author(s) and do not necessarily reflect the position of European Publishing. European Publishing accepts no responsibility or liability for any consequences arising from the use of, or reliance on, this content.

Supplementary 1

No.	Question	Response options
1	Sex?	○ Male<br>○ Female<br>○ Other
2	Year of birth	_________ (calendar year)
3	What year of university study are you currently in?	○ Year 1<br>○ Year 2<br>○ Year 3<br>○ Year 4<br>○ Final year/Other (specify): ___________
4	What is your field/major of study?	___________________________________
5	Who are you currently living with?	○ Both parents<br>○ Mother or father<br>○ Relatives<br>○ In a dormitory/rented accommodation<br>○ Other (specify): ___________
6	Survey location	○ Hanoi<br>○ Ho Chi Minh<br>○ Hue<br>○ Da Nang
7	Have you ever smoked conventional cigarettes?	○ Never<br>○ Ever smoked (even a few puffs or smoked occasionally)<br>○ Currently smoke conventional cigarettes (occasionally or regularly)
8	Do any people around you use conventional cigarettes? (Select all that apply.)	☐ Father ☐ Mother<br>☐ Sibling(s) ☐ Dormitory/roommate(s)<br>☐ Classmate(s) ☐ Close friend(s)<br>☐ No one ☐ Other: ___________
9	Have you ever used an e-cigarette (EC)?	○ Ever tried (even a few puffs or occasional use)<br>○ Currently use (occasionally or regularly)<br>○ Never
10	Have you ever used a heated tobacco product (HTP)?	○ Ever tried (even a few puffs or occasional use)<br>○ Currently use (occasionally or regularly)<br>○ Never
11	Have you ever used an e-cigarette (EC)?	○ Ever tried (even a few puffs or occasional use)<br>○ Currently use (occasionally or regularly)<br>○ Never
12	Have you ever used a heated tobacco product (HTP)?	○ Ever tried (even a few puffs or occasional use)<br>○ Currently use (occasionally or regularly)<br>○ Never
13	Do any people around you use e-cigarettes/HTPs? (Select all that apply.)	☐ Father ☐ Mother<br>☐ Sibling(s) ☐ Roommate(s)<br>☐ Classmate(s) ☐ Close friend(s)<br>☐ No one ☐ Other: ___________

No.	Question	Response options
14	During the past 30 days, have you seen advertising for e-cigarettes/HTPs?	○ Yes<br>○ No
